# Assembly and phylogeographical analysis of novel *Taenia solium* mitochondrial genomes suggest stratification within the African-American genotype

**DOI:** 10.1186/s13071-023-05958-z

**Published:** 2023-10-06

**Authors:** Gabriel Jiménez-Avalos, Alina Soto-Obando, Maria Solis, Robert H. Gilman, Vitaliano Cama, Armando E. Gonzalez, Hector H. García, Patricia Sheen, David Requena, Mirko Zimic, for the Cysticercosis Working Group in Peru

**Affiliations:** 1 Laboratorio de Bioinformática, Biología Molecular y Desarrollos Tecnológicos. Laboratorios de Investigación y Desarrollo, Facultad de Ciencias e Ingeniería. Universidad Peruana Cayetano Heredia, Lima, Perú; 2 Present Address: T. C. Jenkins Department of Biophysics, Johns Hopkins University, Baltimore, MD USA; 3 Department of International Health, Bloomberg School of Public Health, Johns Hopkins University, Baltimore, USA; 4 Division of Parasitic Diseases and Malaria, Center for Global Health, Centers for Disease Control and Prevention, Atlanta, USA; 5 Facultad de Medicina Veterinaria, Universidad Nacional Mayor de San Marcos, Lima, Perú; 6 Departamento de Microbiología, Universidad Peruana Cayetano Heredia, Lima, Perú; 7 Cysticercosis Unit, Instituto Nacional de Ciencias Neurológicas, Lima, Perú; 8 Laboratory of Cellular Biophysics, The Rockefeller University, New York, USA; 9 Bioinformatics Group in Multi-Omics and Immunology, New York, NY 10065 USA

**Keywords:** Phylogenetics, Phylogeography, Haplotypes, Taeniasis, Cysticercosis, Genetics, Genomics, Evolution, Molecular epidemiology, Mitochondrial genome

## Abstract

**Background:**

*Taenia solium* is a parasite of public health concern, causing human taeniasis and cysticercosis. Two main genotypes have been identified: Asian and African-American. Although characterizing *T. solium* genotypes is crucial to understanding the genetic epidemiology of its diseases, not much is known about the differences between *T. solium* mitochondrial genomes from different genotypes. Also, little is known about whether genotypes are further subdivided. Therefore, this study aimed to identify a set of point mutations distributed throughout the *T. solium* mitochondrial genome that differentiate the African-American from the Asian genotype. Another objective was to identify whether *T. solium* main genotypes are further stratified.

**Methods:**

One Mexican and two Peruvian *T. solium* mitochondrial genomes were assembled using reads available in the NCBI Sequence Read Archive and the reference genome from China as a template. Mutations with respect to the Chinese reference were identified by multiple genome alignment. Jensen–Shannon and Grantham scores were computed for mutations in protein-coding genes to evaluate whether they affected protein function. Phylogenies by Bayesian inference and haplotype networks were constructed using cytochrome *c* oxidase subunit 1 and cytochrome *b* from these genomes and other isolates to infer phylogeographical relationships.

**Results:**

A set of 31 novel non-synonymous point mutations present in all genomes of the African-American genotype were identified. These mutations were distributed across the mitochondrial genome, differentiating the African-American from the Asian genotype. All occurred in non-conserved protein positions. Furthermore, the analysis suggested a stratification of the African-American genotypes into an East African and a West African sublineage.

**Conclusions:**

A novel set of 31 non-synonymous mutations differentiating the main *T. solium* genotypes was identified. None of these seem to be causing differences in mitochondrial protein function between parasites of the two genotypes. Furthermore, two sublineages within the African-American genotype are proposed for the first time. The presence of the East African sublineage in the Americas suggests an underestimated connection between East African and Latin American countries that might have arisen in the major slave trade between Portuguese Mozambique and the Americas. The results obtained here help to complete the molecular epidemiology of the parasite.

**Graphical Abstract:**

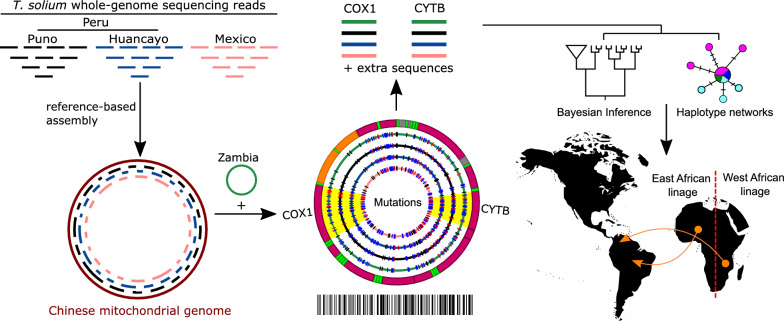

**Supplementary Information:**

The online version contains supplementary material available at 10.1186/s13071-023-05958-z.

## Background

*Taenia solium* is a parasite of public health relevance. Humans are its only known definitive host, harboring the adult tapeworm and releasing infectious eggs into the environment, while domesticated pigs are the usual intermediate host [1–3]. This parasite causes two critical diseases in humans: taeniasis and cysticercosis. The former refers to the intestinal infection with the adult stage of the parasite and is usually caused by eating undercooked pork infected with larval cysts. The latter is the infection with its larvae and mainly occurs due to ingesting food and water contaminated with *T. solium* eggs from the feces of an infected human. Cysticercosis can progress to the central nervous system, causing neurocysticercosis, the leading cause of acquired adult epilepsy in low- and middle-income countries [4]. Because humans are the definitive host, the pattern of *T. solium* spread is driven by human interaction dynamics and behavior [5, 6]. Given the public health importance of *T. solium* studies, assessment of its intraspecific variability and phylogeography is needed to understand its epidemiology, pathogenicity, and transmission [6].

*Taenia solium* has spread globally, being endemic and highly prevalent in Asia, Africa, and Latin America [7]. Interestingly, it has been shown that *T. solium* intraspecific variability in the mitochondrial genome is strongly related to the geographical origin of the specimens [5, 6, 8, 9]. Two main genotypes have been identified: Asian and African-American [5]. This relation has been shaped by human migration and trade [5, 6, 9–13]. For example, the similarity between Latin American, African, and Philippine *T. solium* populations resulted from the gene flow established by the European maritime trade routes of the fifteenth through to the nineteenth centuries [6]. Additionally, the sympatric coexistence of the Asian and African-American genotypes in Madagascar is explained by two independent human groups that migrated to this island and introduced both lineages [9, 10].

The aforementioned geographical genetic variability is expected to result in clinical heterogeneity in *T. solium* diseases between regions [14]. Therefore, an exhaustive study of it is crucial for completing the epidemiology of taeniasis and cysticercosis [5, 15]. Additionally, the geographical variability can be used to assess the impact of human migration and trade on the spread of this parasite [9, 10], which is essential to preventing its dissemination. However, there is a lack of knowledge about differences and similarities between whole *T. solium* mitochondrial genomes from different genotypes due to the low number of assembled genomes available. Likewise, few efforts have been made to identify whether the main *T. solium* genotypes are further stratified. Combining newly assembled sequences and those reported worldwide in phylogeographical studies could help fill this knowledge gap.

So far, cytochrome *c* oxidase subunit 1 (*COX1*) and cytochrome *b* (*CYTB*) have proved to be valuable markers for this purpose [5, 6, 8–12]. Accordingly, they are the genes with the most *T. solium* sequences available from diverse geographical origins. Both have low variability [5]; however, *CYTB* is slightly more variable than *COX1* in *T. solium* [5, 16]. This observation suggests that *CYTB* could be a more suitable marker for *T. solium* intraspecific studies [16]. Considering the currently available data, a comprehensive analysis of multiple sequences from different origins is needed to evaluate whether *CYTB* is more suitable than *COX1* for this kind of analysis.

Due to the high geographical variability in *T. solium*, the present work hypothesizes that a set of common point mutations distributed along the African-American mitochondrial genomes differentiate them from the Asian ones. This work also hypothesizes that further stratification within each genotype might exist. To test this, we assembled and annotated the *T. solium* mitochondrial genomes of two Peruvian and one Mexican isolate. Those genomes, the Chinese reference mitochondrial genome [17], and a genome from Zambia [18] were compared to identify the polymorphisms that characterize the African-American genotype. Finally, the *COX1* and *CYTB* complete sequences from the Latin American isolates and others reported worldwide were included in a phylogeographical reconstruction to analyze further differentiation within each genotype.

## Methods

### Assembly and annotation of the mitochondrial genomes

Unassembled *T. solium* whole-genome sequencing reads from two Peruvian isolates were downloaded from the National Center for Biotechnology Information (NCBI) Sequence Read Archive. They were collected in a previous study conducted by our group [19]. One was from the southern highland city of Huancayo (accession code: SRR644531) and sequenced in the Genome Sequencer FLX (GS FLX) with an average read length of 502 nucleotides and 100× coverage. The second was from the central highland city of Puno (accession code: SRR650708) and sequenced in an Illumina HiSeq 200 with 104-nucleotide-long paired-end reads and 132× coverage. In addition, unassembled whole-genome sequencing reads from a Mexican *T. solium* isolate generated by another laboratory were also downloaded from the same database (accession code: SRR524725). These were sequenced in an Illumina Genome Analyzer IIx with 74-nucleotide-long paired-end reads and 110× coverage. Of note, each sample came from a unique organism.

A reference-based genome assembly strategy was employed to construct the mitochondrial genomes of the *T. solium* samples. Reads for each sample were independently mapped against the *T. solium* reference mitochondrial genome (Chinese isolate, GenBank ID: NC_004022) [17] in the CLC Genomics Workbench v. 21.05.5 (https://digitalinsights.qiagen.com/). Reads that mapped to the reference were exported to a FASTQ file, and their Phred quality scores were obtained with FastQC v. 0.11.9 (http://www.bioinformatics.babraham.ac.uk/projects/fastqc/). Next, the reads that mapped were trimmed using the modified-Mott algorithm implemented in CLC Genomics Workbench v. 21.05.5. To that end, a base-calling probability error limit of 0.001 (equivalent to a Phred quality score limit of 30) was employed. The trimmed reads of each sample were then re-mapped against the reference implemented in the CLC Genomics Workbench. The re-mappings were exported to a binary alignment and map (BAM) file, and its average coverage was computed using the pileup script of BBTools v. 38.91 (http://sourceforge.net/projects/bbmap/).

The consensus sequences were obtained from the re-mapping BAM files using CLC Genomics Workbench v. 21.05.5. Low-coverage regions were defined as positions with less than 5% of total reads supporting them. The ambiguity symbol “N” was inserted in low-coverage regions that presented conflicts (at least one of the reads with a different residue than the rest). However, the most frequent base (voting) was inserted if conflicts occurred in high-coverage regions.

The consensus sequences of each sample were manually curated to correct misassemblies, which resulted in the final assembled genomes being analyzed in further steps. The annotation of the final assembled genomes was performed using CLC Main Workbench v. 21.05.5 using the annotation of the Chinese mitochondrial genome as a reference.

In addition, the same quality-trimmed reads of each sample used to generate the consensus were de novo assembled in CLC Genomics Workbench v. 21.05.5. The purpose of the de novo assembly was to detect gene order rearrangements. Quality control was performed on each contig (identifying chimeric sequences, misassemblies, and artifacts). The depth coverage, the percentage guanine-cytosine (%GC), and the N50 of the assembly were computed. The final de novo contigs of each sample were aligned against the corresponding reference-based assembled genomes to detect potential structural variations.

### Variability and selective pressure

Whole-genome multiple sequence alignment of the assembled Peruvian and Mexican genomes and an already assembled Zambian mitochondrial genome [18] was performed in Mauve v. 2.4.0 [20], using the Chinese *T. solium* mitochondrial genome as reference and default parameters. The resulting alignment was used as input to identify point mutations with respect to the Chinese genome using DnaSP v. 6.12.03 [21]. Point mutations were manually curated to rule out possible sequencing, alignment, or variant calling errors, and were classified into three categories, namely synonymous, non-synonymous, and mutations in non-coding regions based on the mitochondrial genetic code for echinoderms and flatworms [17, 22]. This was performed using DnaSP v. 6.12.03 software and an in-house Python script. Point mutations were graphically represented on a scaled circular map using Circos v. 0.69 [23].

The variability in the 12 protein-coding genes was evaluated as the level of sequence difference (D) [24] for each pairwise combination of the genomes from China, Zambia, Puno, Huancayo, and Mexico. Each of these genes was re-aligned using the web implementation of the EMBOSS needle algorithm [25]. D was computed as D = 1 − (M/L), where M is the number of invariant sites and L is the difference between the alignment length and ambiguous bases.

The pairwise non-synonymous to synonymous substitution (Ka/Ks) ratio of each African-American protein-coding gene against the Chinese reference sequence was computed to estimate and compare the selection pressure of the African-American *T. solium* mitochondrial genomes. For this purpose, the multiple genome alignment was split into 12 protein-coding gene alignments and submitted to the DnaSP software to calculate the ratio.

### Analysis of non-synonymous mutations

The non-synonymous mutations common to all the African-American genomes were analyzed to evaluate whether they might have affected protein function and structure. First, the degree of conservation of these mutations’ positions was assessed using the Shannon–Jensen conservation score [26]. Each protein-coding sequence of the Chinese genome was blasted against the NCBI non-redundant protein sequence database, retrieving a maximum of 5000 hits. Hits with less than 50% identity and coverage and greater than 0.01 expected value were discarded. A multiple sequence alignment between the hits and the corresponding Chinese protein-coding sequence was then generated in the software MAFFT v. 7 [27, 28] using the progressive G-INS-1 method. The resulting alignment was employed as input for the score conservation algorithm developed by Capra and Singh [26] to calculate the Jensen–Shannon conservation scores. The positions were then ranked from highest (conserved) to lowest (not conserved) score, and the top 30 were considered conserved and, therefore, probably functionally/structurally important [26]. Finally, the top 30 of each protein-coding gene were examined to determine whether it included any of the common non-synonymous mutations found in this study.

In addition, the level of physicochemical variation between the original and the mutated amino acid was assessed through the Grantham score [29]. Substitutions with a Grantham score higher than 60 were considered to change the amino acid's physicochemical nature (not conservative) [30].

### Phylogenetic analysis

Complete sequences of *COX1* and *CYTB* mitochondrial genes from the genomes assembled and from different isolates reported worldwide were independently employed to perform two phylogenetic reconstructions. This approach was followed to determine whether both genes supported a similar evolutionary history.

The database comprised a total of 45 *T. solium* *COX1* and 31 *CYTB* complete sequences available in GenBank, including, as the outgroup, sequences from *Taenia saginata* (*COX1*: AB066495.1 and NC_009938.1; *CYTB*: AB066581.1 and NC_00938. 1), *Taenia asiatica* (*COX1*: AB066494.1 and NC_004826.2; *CYTB*: AB066580.1 and NC_004826.2), and *Echinococcus multilocularis* (*COX1* and *CYTB*: NP_000928.2).

Global multiple sequence alignments were performed independently for *COX1* and *CYTB* in MAFFT v. 7 software [27, 28] using the progressive G-INS-1 method. Misaligned regions due to ambiguous nucleotides were trimmed using the Gblocks server v. 0.91 [31, 32] with default options.

Phylogenetic analysis was conducted separately for *COX1* and *CYTB* by maximum likelihood (ML) and Bayesian inference (BI). ML was conducted in RaxML v. 8.2.12 [33]. The GTRCAT evolutionary model (a reversible model of eight parameters) was used, and 1000 bootstrap replicates were performed to estimate the branch robustness.

BI was conducted in BEAST2 v. 2.6.1 [34], implemented on the CIPRES online server platform [35]. The evolutionary model was estimated with jModelTest2 v. 2.1.6 [36] for *COX1* (GTR+G with four gamma categories) and *CYTB* (GTR+I, I = 0.5720), using the corrected Akaike information criterion (AICc) [9, 13]. The analysis was run for 50 million generations, sampling every 5000 generations and using a burn-in of 10% to obtain an effective sample size (ESS) greater than 200. Lastly, a maximum clade credibility tree (MCC) was generated in TreeAnnotator v. 2.6.0 [34].

### Haplotype network

Haplotypes were identified in DnaSP v. 6.12.03 using as input the complete multiple alignments of *COX1* and *CYTB* previously generated (before Gblocks trimming) and considering the total number of point mutations as nucleotide substitutions. The haplotype networks were calculated by median joining [37] using Networks v. 10 software (fluxus-engineering.com). The genetic differentiation (ɸ_ST_) between the African-American subclades 1 and 2 was calculated using a haplotype distance matrix in Arlequin v. 3.5.2.2 [38].

## Results

### Genome assembly and annotation

Each genome was assembled following a reference-based strategy. To that end, the reads from each sample were trimmed by quality. Phred quality scores before trimming were greater than Q30 (Additional file 1: Table S1) for all samples. Quality-trimmed reads from the Peruvian and Mexican isolates were then mapped against the Chinese reference. This resulted in 1,317,941 mapped reads from Puno (7811× coverage), 5561 from Huancayo (42×), and 674,666 from Mexico (3395×). The consensus sequences from each set of mapped reads were extracted and manually curated, resulting in the final assembled genomes being analyzed further.

Genomes from Puno and Mexico were complete (no ambiguous nucleotides present), while the one corresponding to Huancayo was partial (ambiguous nucleotides present). The three genome sequences were of similar length (13,700–13,709 nucleotides). The size of the protein-coding genes in Latin American samples was identical to the Chinese reference (Additional file 1: Table S2). An exception occurred only for *CYTB* in the isolate from Huancayo, which has a missing codon corresponding to positions 872–874 of the Chinese reference.

The reference-based genome assembly followed will bias the order of the genes to be as in the Chinese reference, hiding potential gene structural rearrangements. Therefore, the same quality-trimmed reads used to generate the consensus sequence of each sample were de novo assembled. As a de novo assembly does not require a reference, the gene position within contigs generated by this procedure is not biased, making it more sensitive to detect rearrangements. The de novo assembled contigs perfectly matched the corresponding regions in the reference-based assembled genomes, suggesting no structural rearrangements.

### Variability and selective pressure analysis

**Fig. 1:**
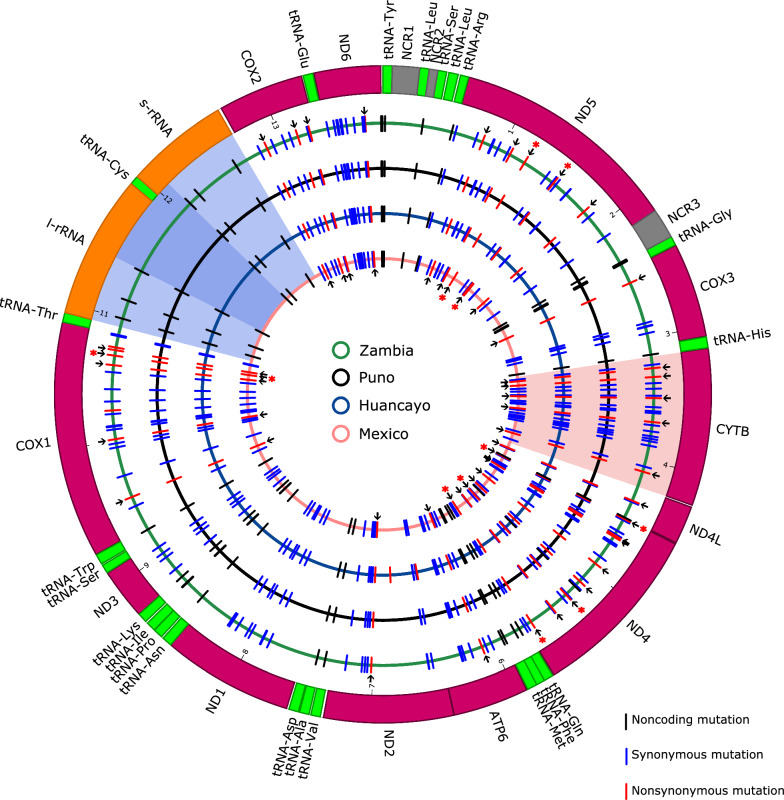
Nucleotide and amino acid differences in *T. solium* genomes with respect to the Chinese reference. The thick outer circle depicts the Chinese *T. solium* reference mitochondrial genome (NCBI Reference Sequence: NC_004022.1), where inner boxes represent the coding sequences (CDS). The color code represents the CDS type: purple for protein-coding genes, green for transfer RNAs (tRNAs), orange for ribosomal RNAs, and gray for non-coding regions (NCR). The inner circles represent the three *T. solium* mitogenomes assembled in this study (black: Peru–Puno, blue: Peru–Huancayo, and pink: Mexico) and the genome from Zambia. Blue, red, and black bars indicate synonymous, non-synonymous mutations, and mutations in non-coding regions, respectively. Flanking arrows highlight non-synonymous substitutions that were present in all the African-American genomes analyzed. An asterisk above some arrows indicates that the mutation involved a change in the amino acid nature, as revealed by Grantham scores higher than 60. The circular segments shaded in transparent blue and red indicate low and high variability regions, respectively. Darker blue within the blue-shaded circular segment indicates a region fully conserved in the four mitochondrial genomes. Mutations in the low variability region suggest that the region could differentiate Asian isolates from African-American isolates but not between African-American samples

Mutations with respect to the Chinese reference mitochondrial genome, D, and pairwise Ka/Ks ratios per protein-coding gene were computed to obtain a detailed comparison between whole mitochondrial genomes of different genotypes. The distribution of point mutations in the African-American genomes was almost identical (Fig. 1). Of the 36 non-synonymous mutations, 31 were present in all African-American samples (same mutated nucleotide in all African-American genomes) (Fig. 1, Table 1). They were distributed in all protein-coding genes except for NADH-ubiquinone oxidoreductase chain 1 (*ND1*) and NADH-ubiquinone oxidoreductase chain 3 (*ND3*).

**Table 1:** Non-synonymous point mutations present in all African-American mitochondrial genomes with respect to the Chinese reference

Protein-coding gene	Amino acid substitution
*ATP6*	V59I (0.53, 29)
*COX1*	S90G (0.46, 56), I253V (0.67, 29), V454M (0.48, 21), C480R (0.36, 180), N495D (0.34, 23)
*COX2*	V56I (0.61, 29), L145I (0.68, 5), G180S (0.64, 56)
*COX3*	V35M (0.39, 21)
*CYTB*	V33I (0.51, 29), I57V (0.58, 29), V114L (0.64, 32), V179I (0.61, 29), I316V (0.43, 29)
*ND2*	S192N (0.54, 46)
*ND4*	V3A (0.30, 64)*, S54N (0.32, 46), S59G (0.26, 56), N147E (0.25, 42), N211D (0.50, 23), S263P (0.43, 74)*, I315V (0.49, 29), H383Y (0.39, 83)*
*ND4L*	M39V (0.57, 21)
*ND5*	E82K (0.52, 56), I164V (0.48, 29), A209S (0.62, 99)*, S305C (0.66, 112)*, D418N (0.31, 23)
*ND6*	V109I (0.44, 29)

Amino acid substitutions present in all the assembled African-American genomes are given per protein-coding gene. The Jensen–Shannon conservation and the Grantham score for each substitution are shown in parentheses. High Jensen–Shannon and Grantham scores indicate conserved positions and substitutions with a radically different amino acid, respectively. The threshold for classifying a position as conserved using the Jensen–Shannon score is calculated based on the top 30 positions with the highest scores in the protein sequence (see materials and methods). Threshold values per protein-coding gene are 0.74 (*ATP6*), 0.81 (*COX1*), 0.76 (*COX2*), 0.77 (*COX3*), 0.76 (*CYTB*), 0.73 (*ND1*), 0.77 (*ND2*), 0.73 (*ND3*), 0.8 (*ND4*), 0.74 (*ND4L*), 0.81 (*ND5*), and 0.74 (*ND6*). None of the non-synonymous mutations passed the conservation threshold. Substitutions with a Grantham score higher than 60 were considered to affect the physicochemical nature of the amino acid. These mutations are shown with an asterisk (*)

**Fig. 2:**
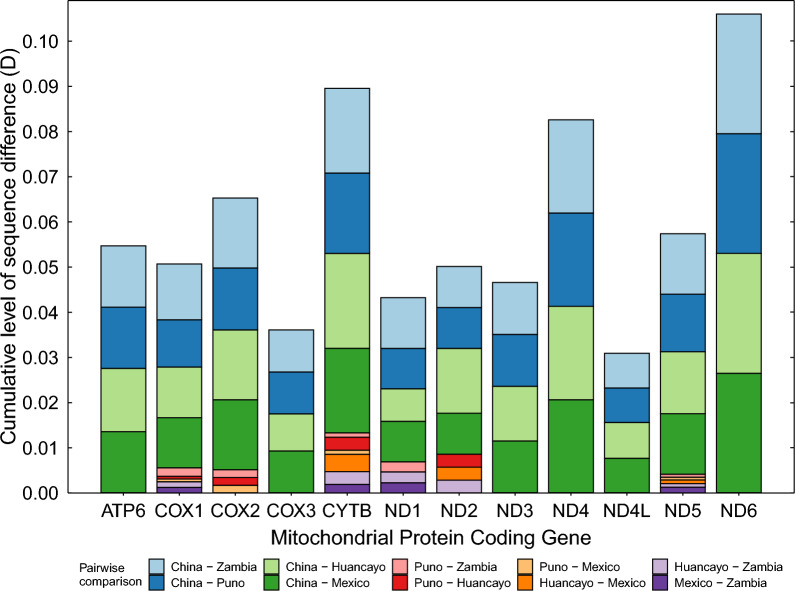
Level of sequence difference (D) per protein-coding gene of each possible pairwise combination of four mitochondrial genomes (Chinese reference, Peru–Huancayo, Peru–Puno, and Mexico). D values were computed by performing all possible pairwise alignments and applying the formula D = 1 − (M/L), where L is the difference between the alignment length and the number of ambiguous codons, and M is the number of invariant sites in the alignment. Different D values for each combination were stacked and presented per protein-coding gene in a bar plot. Thus, the height of a particular bar of a gene corresponds to the sum of D values for the different pairwise combinations. In other words, the bar height is a cumulative D value

The similarity in the point mutation distribution among African-American samples is also supported by the small contribution of Zambia, Puno, Huancayo, and Mexico pairwise comparisons in the cumulative D (Fig. 2). This was less than 0.015 for *CYTB*; less than 0.01 for *COX1*, cytochrome *c* oxidase subunit 2 (*COX2*), NADH-ubiquinone oxidoreductase chain 2 (*ND2*), and NADH-ubiquinone oxidoreductase chain 5 (*ND5*); and 0 (sequences were identical) for the other protein-coding genes (Fig. 2).* CYTB* was the only one with non-zero values for all pairwise comparisons. *COX1* and *ND5* had zero values for Huancayo–Mexico and Puno–Huancayo pairwise comparisons, respectively, and non-zero values for the rest. Five genes showed relatively high D values: ATP synthase subunit 6 (*ATP6*), *COX2*, *CYTB*, NADH-ubiquinone oxidoreductase chain 4 (*ND4*), *ND5*, and NADH-ubiquinone oxidoreductase chain 6 (*ND6*).

**Fig. 3:**
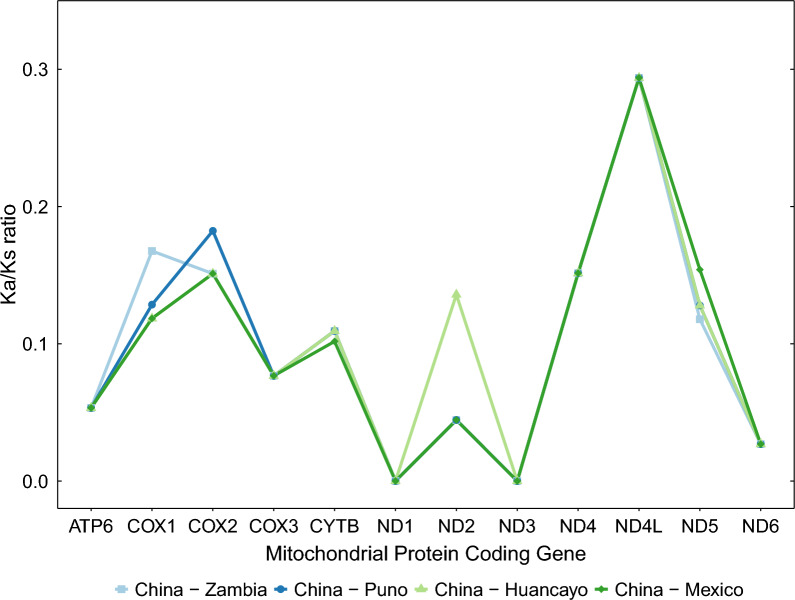
Substitution ratios per protein-coding genes of the *T. solium* mitochondrial genomes. The pairwise ratios (Ka/Ks) of non-synonymous (Ka) over synonymous substitution rates (Ks) are shown per protein-coding gene for each of the African-American mitochondrial genomes presented in this work. Pairwise Ka/Ks ratios were computed between African-American and Chinese reference sequences. Note that *ND1* and *ND3* pairwise Ka/Ks ratios are equal to 0 due to the absence of non-synonymous mutations

Remarkably, a similar pattern of pairwise Ka/Ks ratios was observed in all African-American samples. In fact, the African-American samples had the same pairwise Ka/Ks values for *ATP6*, cytochrome *c* oxidase subunit 3 (*COX3*), *ND4*, NADH-ubiquinone oxidoreductase chain 4L (*ND4L*), and *ND6*. All pairwise Ka/Ks ratios were less than 1 for all the protein-coding genes (Fig. 3). In particular, *ND1* and *ND3* seem to be subject to an absolute purifying selection (Ka/Ks = 0) in all the samples evaluated.

### Functionality assessment of the common non-synonymous mutations

Jensen–Shannon conservation and Grantham scores were assigned to the 31 common non-synonymous mutations to assess whether they occurred in conserved positions and involved a radical change in the physicochemical properties of the position, respectively (Table 1). Radical mutations in conserved positions potentially affect protein function. Six of the 31 non-synonymous mutations changed the physicochemical nature of the amino acid, as revealed by Grantham scores higher than 60. These were located within *COX1*, *ND4*, and *ND5* (Fig. 1, Table 1). Nevertheless, none of the positions of the 31 mutations passed the conservation threshold of the present study (see materials and methods and Table 1 legend), meaning they are unlikely to affect protein function and structure despite altering local physicochemical properties.

### Phylogenetic analysis

The fact that the sequences of *CYTB*, *COX1*, and *ND5* of the African-American genomes had differences between them raised the question of whether the African-American genotype is further subdivided. To test this, two phylogenetic reconstructions using *COX1* and *CYTB* individually were performed, including the isolates of this study and others reported worldwide. This approach was followed to determine whether consensus exists in the evolutionary history of phylogenies constructed with both markers.

The phylogenetic analysis distinguished two major clades for both markers: Asian and African-American. Both genes supported the Asian (*COX1*: posterior probability [PP] = 0.95; *CYTB*: bootstrap [BS] = 96%, PP = 1.0) and African-American (*COX1*: BS = 98%, PP = 0.99; *CYTB*: BS = 98%, PP = 0.99) clades. However, a sequence from Tanzania was not included in the *CYTB* African-American clade.

**Fig. 4:**
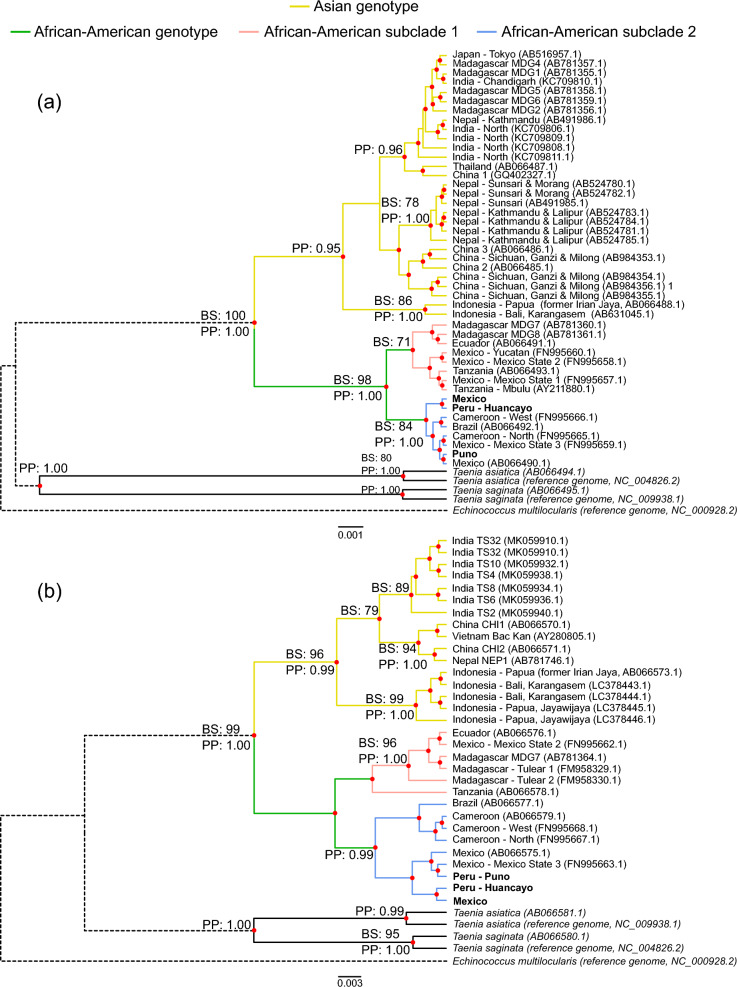
Phylogenetic trees constructed from *COX1* and *CYTB* complete gene sequences. **a** Bayesian inference (BI) tree using *COX1* complete gene sequences. **b** Same as **a** but for *CYTB*. Posterior probabilities higher than 0.95 are shown for selected groups. Maximum likelihood (ML) trees were also constructed with the same sequences (not shown). Bootstrap values (BS) higher than 70% are shown for the selected groups that appeared in the BI and ML trees

For *COX1*, the Asian group included three supported subclades (Fig. 4a). The first comprised countries from East (China and Japan), South (India and Nepal), and Southeast Asia (Thailand), along with the island of Madagascar (PP: 0.96). The second included sequences from Nepal (BS: 78, PP: 1.00), which were directly related to an unsupported group of Chinese sequences. The third comprised two sequences from Indonesia (BS: 86, PP: 1.00). In the *CYTB*-based tree (Fig. 4b), groups similar to the second (BS: 94, PP: 1.0) and third (BS: 99, PP: 1.0) subclades were also supported. In addition, a group formed by just Indian samples was present in the *CYTB* phylogeny (BS: 89), which might be equivalent to the first subclade.

In the African-American genotype, two subclades appeared within the *COX1* phylogeny. The first (African-American subclade 1; BS: 71) consisted of samples from Tanzania and Mexico (Yucatan, Mexico State 1 and 2). Of note, this group only included sequences from East Africa (Tanzania and Madagascar). The second (African-American subclade 2; BS: 84 and PP: 1.0) comprised samples from Mexico (Mexico State 3 and the Mexican sequence assembled in this study), Cameroon (West and North), Peru (Puno and Huancayo), and Brazil. Of note, this group only had sequences from West Africa (Cameroon). *CYTB* presented a similar topology for the African-American genotype as that obtained with *COX1*.

### Haplotype network

To confirm whether the samples of the subclades identified formed differentiated subpopulations, haplotype networks with *COX1* and *CYTB* were constructed using the complete multiple alignments employed in the phylogenetic reconstruction (before Gblocks extraction). As mentioned above, only *CYTB* alignment presented positions with ambiguous nucleotides (in the sequence from Huancayo), and those were excluded for the haplotype network construction to avoid adding ambiguity. The exclusion resulted in an alignment of 1044 positions (97.8% of the total *CYTB* length).

**Fig. 5:**
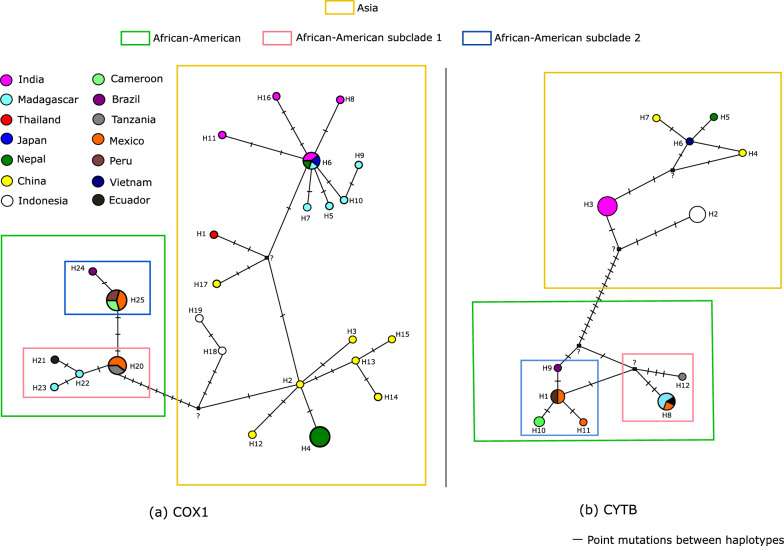
Haplotype network of *COX1* and *CYTB*. **a** *COX1* network. **b** *CYTB* network. The geographical origins of the samples included in each haplotype are color-coded. Colored squares enclose the most important clades and subclades

From the multiple alignment of 45 *COX1* sequences, 43 positions of high variability were identified, supporting 25 haplotypes. These were diagrammed according to their genetic distances in a haplotype network (Fig. 5). Sequences comprising each haplotype are listed in Additional file 1: Table S4. In contrast, the alignment of 31 *CYTB* sequences collapsed just into 12 haplotypes, which were generated from 30 polymorphic sites. The haplotype diversity was 0.937 for *COX1* and 0.893 for *CYTB*, respectively.

Except for the Indonesian samples, both haplotype networks suggested that the Asian samples were closely related, with no resolution to support their stratification into subclades. In the *COX1* network (Fig. 5a), haplotype 6 (H6) was the main dispersion center, directly connected to haplotypes composed by sequences from India (H11, H16, H8) and Madagascar (H5, H7, H9, H10). One of its branches connected to an unknown haplotype, which diverged into a haplotype from Thailand (H1) and China (H17). The unknown haplotype was also linked to the Chinese haplotype H2, from which other Chinese and Nepali haplotypes diverged. For *CYTB* (Fig. 5b), the main dispersion center (H3) connected to Chinese (H4 and H7), Nepali (H5), and Vietnamese (H6) samples through an unknown haplotype. Indonesian samples remained isolated from the rest of Asian countries in both networks, forming another subpopulation. For *COX1*, the isolate from Papua (former Irian Jaya, H18) seemed more basal than that from Bali (H19). For the *CYTB* network, all Indonesian samples were clustered together (H2).

Both haplotype networks distinguished two groups related to the African-American subclades 1 and 2. Based on the *COX1* haplotype network, African-American subclade 1 seems closer to the Asian genotype. To determine whether the isolates that formed African-American subclade 1 were genetically different from those of African-American subclade 2, a computation of the ɸ_ST_ value between these two groups was made. Values were 0.83 for *COX1* and 0.62 (*P* < 0.05) for *CYTB*.

## Discussion

Two main *T. solium* genotypes have been identified, the Asian and the African-American [5]. However, a detailed whole-genome comparison between mitochondrial genomes from each genotype is still missing. The present study fills that knowledge gap by assembling the mitochondrial genomes from three African-American *T. solium* isolates from Peru–Puno, Peru–Huancayo, and Mexico and comparing them against the Chinese reference mitochondrial genome from the Asian genotype. This allows the identification of novel differences between the genotypes at the whole-mitochondrial-genome level.

The mitochondrial genomes from Puno-Peru and Mexico had 7,811X and 3,395X of coverage, respectively. They had no ambiguous nucleotides and were the same size as the reference mitochondrial genome from China. The genome from Huancayo-Peru had a lower coverage (42×) and, despite being partial (ambiguous nucleotides present), was still adequate for the rest of the analysis. As expected, the genome size and the %GC are similar between these three isolates, supporting the assembly method. No structural variations with respect to the Chinese reference were detected (Fig. 1, Additional file 1: Table S2). The size of each protein-coding gene is the same, except for the gene *CYTB* in Huancayo, which has one codon less (Additional file 1: Table S2). As in the Chinese reference, no evidence of an extended non-coding control region (such as the one in *Echinococcus granulosus* [39]) was observed in any of the three genomes. Nakao et al. [17] previously reported the presence of an abbreviated stop codon U (or T in DNA) at the *ND1* gene. Notably, all the mitochondrial genomes assembled in the present study present this stop codon, supporting this observation.

At least 160 point mutations with respect to the Chinese reference were identified in each of the four African-American genomes (Fig. 1). This suggests differentiation between Asian and African-American genotypes encoded throughout the mitochondrial genome. Notably, the differentiation is not limited to specific genes. In addition, the point mutation distribution is almost identical across genomes of the African-American genotype and concentrated in protein-coding genes. This agrees with the fact that their protein-coding gene sequences match well, as revealed by their low pairwise D values (Fig. 2), and that their protein-coding genes are subjected to similar selective pressure when compared to the Chinese ones (Fig. 3).

The similarity between African-American samples and their marked differentiation with respect to the Chinese reference supports the possibility of identifying common mutations distributed throughout their mitochondrial genomes that differentiate them from the Asian isolates. Indeed, 31 non-synonymous mutations are present in all the African-American genomes (same nucleotide or "allele" present in all African-American samples) (Fig. 1, Table 1). Those may represent a starting point for identifying a molecular signature to classify an isolate into one of the two genotypes.

Jensen–Shannon conservation and Grantham scores were employed to test whether any of the 31 non-synonymous mutations might account for differences in mitochondrial protein function between parasites of the two genotypes. A high value of both scores indicates mutations at conserved positions that radically affect the local physicochemical properties of the protein and are, therefore, likely to affect its function. None of the mutations’ positions passed the conservation threshold of the present study (Table 1). This means that they are unlikely to cause differences in the mitochondrial protein function and structure of African-American parasites compared to the Asian ones. 

The present results suggest that *CYTB* is the most variable mitochondrial gene. It has the highest density of mutations (Fig. 1), the highest cumulative D, and different D values for all pairwise comparisons (Fig. 2). This result agrees with previous studies [5, 16]. For instance, 28 single-nucleotide polymorphisms (SNPs) (1.7% variability rate) in the *COX1* gene were found in contrast to the 31 SNPs (2.9% variability rate) present in *CYTB* [5]. Despite this, *CYTB*-based haplotype networks constructed here had fewer haplotypes and lower haplotype diversity than those constructed with *COX1*. Nevertheless, it must be considered that there are more complete sequences for *COX1* than for *CYTB*. Accordingly, the database used in the present study comprised 45 *COX1* and 31 *CYTB* sequences. The smaller sampling likely explains why the *CYTB*-based network has lower haplotype diversity than the *COX1* network.

Because of its variability, it has been stated that *CYTB* is a better marker for reconstructing phylogenies among closely related groups (such as intraspecific variations) within Taeniidae [16]. However, because of the different number of *COX1* and *CYTB* sequences used here, the present work cannot compare the phylogenies of both genes to confirm this statement. Nevertheless, given the smaller number of *CYTB* sequences, the lower haplotype diversity suggests that the higher variability of *CYTB* cannot compensate for the smaller number of complete sequences available. In other words, with the data available so far, *COX1* may be a better choice for inferring phylogeographical relationships if complete sequences are to be used.

*ATP6*, *COX2*, and *ND6* also have a relatively high D value; however, these genes have a short length. D values could overestimate the variability for small genes, as the percentage of identity is inversely correlated with the alignment length. Thus, high D values for small sequences as these three should be taken cautiously and do not necessarily imply high variability.

This study suggests that *ND1* and *ND3* are currently subjected to a strong purifying selection in the four African-American genotypes (Ka/Ks = 0) (Fig. 3). In that sense, mutations in these genes seem deleterious and, therefore, negatively selected. The fact that the four African-American genomes had non-synonymous mutations distributed in all protein-coding genes but *ND1* and *ND3* while, at the same time, these two genes displayed synonymous mutations (Fig. 1) suggests that the null Ka/Ks values observed were not a coincidence. Interestingly, *ND1* is a crucial subunit of the mitochondrial respiratory complex I because it allocates the entrance of the quinone reaction chamber and the first half part of the first proton translocation channel, which receives input from the cytoplasm [40, 41]. Moreover, *ND3* provides the flexibility needed for a concerted rearrangement that generates the driving force for proton pumping [42]. Hence, mutations in these genes might affect the quinone reductase activity and collapse the proton translocation system on the inner mitochondrial membrane. The importance of both subunits in *T. solium* metabolism might relate to the fact that this work's data suggest low amino acid variability for these genes. Further sampling will be required to strengthen the presented observations.

Both genes' phylogenetic analysis and haplotype networks showed two main lineages: the Asian and the African-American (Figs. 4 and 5). This has been reported by other studies [5, 8, 11].

In the *COX1* haplotype network (Fig. 5a), samples from Japan, India, Nepal, and Madagascar are grouped in the same haplotype (H6). Considering that *T. solium* is not endemic in Japan, its relation with H6 samples is likely the result of a recent reintroduction, a not-so-rare event in the last years [43]. The Madagascan isolates' close association with Nepali and Indian samples suggests that the parasite was introduced into Madagascar from the Indian subcontinent [10].

A particular case occurred with Nepali samples. While one group was included in H6, as mentioned above, the other was included in H4 in close association with Chinese sequences. These two genetic subpopulations suggest the existence of two gene flows toward Nepal, one from the north (from China), and another from the south (from India). They remain separated, possibly due to the geographical barrier that the Himalayas constitute.

Phylogenetic trees and haplotype networks grouped Indonesian samples in haplotypes that remain distant from other Asian sequences (Figs. 4 and 5). Indeed, *T. solium* subpopulations in Indonesia are isolated, as shown by the fact that this parasite is mainly restricted to Bali and Papua (former Irian Jaya) [12], although sporadic *T. solium* infections also occur in Nusa Tenggara Timur [44]. The *COX1* haplotype network suggested that the sample from Papua is more ancient than the one from Bali because it is closer to other Asian isolates. Nonetheless, this proposal seems inconsistent with the epidemiological evidence that suggests that the introduction of *T. solium* to Papua was made 50 years ago from Bali [12, 45]. Interestingly, although *CYTB* is more variable than *COX1*, the *CYTB* network shows no resolution to distinguish between Indonesian haplotypes, while the *COX1* network does. This inconsistency might suggest that the haplotype differentiation seen in the *COX1* network was an artifact attributed to a random selection when *T. solium* was introduced into Papua, as has been hypothesized in other work [12].

Remarkably, phylogenies and haplotype networks constructed in this work suggest that the African-American lineage is further subdivided into an East African (African-American subclade (1) and a West African sublineage (African-American subclade (2) based on their geographical composition. The genetic differentiation between the two is confirmed by the fact that ɸ_ST_ values were high and significant (*P* < 0.05). The haplotype networks further confirmed the differentiation between sublineages, allowing one to visualize two clusters.

Interestingly, haplotype networks presented in a previous study by Solano et al. [8] also showed that *COX1* sequences from Latin American isolates split into two groups. The study included only one African sequence that was part of one of the groups. The other group was closely related to Asian samples and appeared unrelated to the African sequence. Therefore, Solano et al. concluded that the two groups they observed represented the African American and Asian subclades. However, using a single African sequence makes it impossible to confirm that the group that did not include it does not have African ancestry and thus represents the Asian genotype. It is much more plausible that the two groups observed by Solano et al. actually represent the East and West African sublineages. In agreement, the single African sequence included in one of the groups of Solano et al. is from West Africa, whereas the closer association of the remaining group with Asian samples is also displayed by the East African lineage observed in the present study (Fig. 5a). In any case, the split of sequences from the African-American genotype observed by two independent studies supports the idea of further stratification within this group and calls for further studies to confirm the hypothesis.

The relationship between African and Latin American *T. solium* isolates results from the conversion between the Trans-Atlantic slave and trade routes, which introduced infected slaves and/or pigs from the former to the latter [6]. Although these routes mainly involved West African countries, the presence of the East African lineage in the Americas found in this study suggests that the trade routes also established an important connection between East African and Latin American countries. This connection probably reflects the major slave trade between Portuguese Mozambique and the Americas from 1643 onwards, as this trade also significantly introduced East African sequences into the human mitochondrial DNA pool of the Americas [46].

Notably, isolates from Mexico are from both the East African and West African lineages, which agrees with the proposal that at least two genetic subpopulations are present in that country [11]. In contrast, the isolates from Peru are only from the West African lineage. This is coherent with the fact that almost all enslaved people who arrived in Peru were from West or Southwest Africa [47]. However, studies with a bigger sample size of Peruvian samples should be conducted to support the exclusive presence of the West African lineage in Peru.

## Conclusions

In conclusion, 31 non-synonymous point mutations present in all African-American genomes were identified. Those differentiated the African-American genotype from the Asian genotype. Further analysis is required to test whether they could be used to classify an isolate into one of the two genotypes. None of these mutations occurred in conserved protein positions, suggesting that they are not linked to changes in protein function between parasites of the two genotypes. Strikingly, all phylogeographical analyses showed that the African-American genotype is subdivided into an East African and a West African sublineage. The presence of the East African lineage in the Americas suggests an underestimated connection between East African and Latin American countries that might have arisen from the slave trade between Portuguese Mozambique and the Americas. In summary, the present study shows that a detailed comparison of the mitochondrial variability of *T. solium* still reveals interesting evolutionary features that could be used to combat *T. solium* diseases.

### Supplementary Information

**Additional file 1: Table S1.** Mapping statistics of the genome assembly. The number of reads mapped to the Chinese mitochondrial genome (reference) and their mean quality scores before trimming (pre) are given. The number of reads mapped after trimming (post); and the coverage, the genome length in base pairs (bp), the %GC, and the N50 of the assembly in nucleotides (nt) are also shown. **Table S2.** Gene arrangement of *T. solium* mitochondrial genomes from Peru, Mexico, and China (reference). The size of each genome in base pairs (bp) is given in parenthesis next to the mitochondrial genome name. Position intervals per gene are shown. The size of each gene (in bp) is specified in parentheses next to each position interval. Start and stop codons of each protein-coding gene per mitochondrial genome are also specified. **Table S3.** Sequence composition of the haplotypes formed in the *COX1* and *CYTB* networks. Sequences (with their accession numbers) included in each haplotype are specified.

## Data Availability

The assembled and annotated mitochondrial genomes from Puno and Huancayo were uploaded to GenBank with accession numbers OM967033 and KT591612, respectively. Regarding the assembled genome from Mexico, nucleotide sequence data reported are available in the Third-Party Annotation Section of the DNA Data Bank of Japan (DDBJ)/European Nucleotide Archive (ENA)/GenBank databases under the accession number TPA: BK061219. Other raw data will be available upon request.

## Abbreviations

D: Level of sequence difference
ML: Maximum likelihood
BI: Bayesian inference
ATP6: ATP synthase subunit 6
COX1: Cytochrome *c* oxidase subunit 1
COX2: Cytochrome *c* oxidase subunit 2
COX3: Cytochrome *c* oxidase subunit 3
CYTB: Cytochrome *b*
ND1: NADH-ubiquinone oxidoreductase chain 1
ND2: NADH-ubiquinone oxidoreductase chain 2
ND3: NADH-ubiquinone oxidoreductase chain 3
ND4: NADH-ubiquinone oxidoreductase chain 4
ND4L: NADH-ubiquinone oxidoreductase chain 4L
ND5: NADH-ubiquinone oxidoreductase chain 5
ND6: NADH-ubiquinone oxidoreductase chain 6

## References

[1] Yoshino K (1933). Studies on the post-embryonal development of *Taenia*
*solium* Part I. On the hatching of the eggs of *Taenia*
*solium*. J Formos Med Assoc.

[2] Yoshino K (1933). Studies on the postembryonal development of *Taenia solium.* Part II. On the youngest form of *Cysticercus cellulosae* and on the migratory course of the Oncosphaera of *Taenia solium* within the intermediate host. J Formos Med Assoc.

[3] Yoshino K (1933). Studies on the postembryonal development of *Taenia solium*. Part III. On the development of *Cysticercus cellulosae* within the definite intermediate host. J Formos Med Assoc.

[4] Singh G, Burneo JG, Sander JW (2013). From seizures to epilepsy and its substrates: Neurocysticercosis. Epilepsia.

[5] Nakao M, Okamoto M, Sako Y, Yamasaki H, Nakaya K, Ito A (2002). A phylogenetic hypothesis for the distribution of two genotypes of the pig tapeworm *Taenia solium* worldwide. Parasitology.

[6] Martinez-Hernandez F, Jimenez-Gonzalez DE, Chenillo P, Alonso-Fernandez C, Maravilla P, Flisser A (2009). Geographical widespread of two lineages of *Taenia solium* due to human migrations: can population genetic analysis strengthen this hypothesis?. Infect Genet Evol.

[7] WHO (2015). WHO estimates of the global burden of foodborne diseases: foodborne disease burden epidemiology reference group 2007–2015.

[8] Solano D, Navarro JC, León-Reyes A, Benítez-Ortiz W, Rodríguez-Hidalgo R (2016). Molecular analyses reveal two geographic and genetic lineages for tapeworms, *Taenia solium* and *Taenia saginata*, from Ecuador using mitochondrial DNA. Exp Parasitol.

[9] Michelet L, Carod J-F, Rakontondrazaka M, Ma L, Gay F, Dauga C (2010). The pig tapeworm *Taenia solium*, the cause of cysticercosis: biogeographic (temporal and spacial) origins in Madagascar. Mol Phylogenet Evol.

[10] Yanagida T, Carod J-F, Sako Y, Nakao M, Hoberg EP, Ito A (2014). Genetics of the pig tapeworm in Madagascar reveal a history of human dispersal and colonization. PLoS ONE.

[11] Michelet L, Dauga C (2012). Molecular evidence of host influences on the evolution and spread of human tapeworms. Biol Rev.

[12] Yanagida T, Swastika K, Dharmawan NS, Sako Y, Wandra T, Ito A (2021). Origin of the pork tapeworm *Taenia solium* in Bali and Papua, Indonesia. Parasitol Int.

[13] Hoberg EP, Alkire NL, Queiroz AD, Jones A (2001). Out of Africa: origins of the *Taenia* tapeworms in humans. Proc R Soc Lond B Biol Sci.

[14] Ito A, Budke CM (2021). Genetic diversity of *Taenia solium* and its relation to clinical presentation of cysticercosis. Yale J Biol Med.

[15] Campbell G, Garcia HH, Nakao M, Ito A, Craig PS (2006). Genetic variation in *Taenia solium*. Parasitol Int.

[16] Okamoto M, Nakao M, Sako Y, Ito A (2001). Molecular variation of *Taenia solium* in the world. Southeast Asian J Trop Med.

[17] Nakao M, Sako Y, Ito A (2003). The mitochondrial genome of the tapeworm *Taenia solium*: a finding of the abbreviated stop codon U. J Parasitol.

[18] Sadlowski H, Schmidt V, Hiss J, Kuehn JA, Schneider CG, Zulu G (2021). Diagnosis of *Taenia solium* infections based on "mail order" RNA-sequencing of single tapeworm egg isolates from stool samples. PLoS Negl Trop Dis.

[19] Pajuelo MJ, Eguiluz M, Dahlstrom E, Requena D, Guzmán F, Ramirez M (2015). Identification and characterization of microsatellite markers derived from the whole genome analysis of *Taenia solium*. PLoS Negl Trop Dis.

[20] Darling ACE, Mau B, Blattner FR, Perna NT (2004). Mauve: multiple alignment of conserved genomic sequence with rearrangements. Genome Res.

[21] Rozas J, Ferrer-Mata A, Sánchez-DelBarrio JC, Guirao-Rico S, Librado P, Ramos-Onsins SE (2017). DnaSP 6: DNA sequence polymorphism analysis of large data sets. Mol Biol Evol.

[22] Nakao M, Sako Y, Yokoyama N, Fukunaga M, Ito A (2000). Mitochondrial genetic code in cestodes. Mol Biochem Parasitol.

[23] Krzywinski M, Schein J, Birol İ, Connors J, Gascoyne R, Horsman D (2009). Circos: an information aesthetic for comparative genomics. Genome Res.

[24] Lavikainen A, Haukisalmi V, Lehtinen MJ, Henttonen H, Oksanen A, Meri S (2008). A phylogeny of members of the family *Taeniidae* based on the mitochondrial cox1 and nad1 gene data. Parasitology.

[25] Madeira F, Park YM, Lee J, Buso N, Gur T, Madhusoodanan N (2019). The EMBL-EBI search and sequence analysis tools APIs in 2019. Nucleic Acids Res.

[26] Capra JA, Singh M (2007). Predicting functionally important residues from sequence conservation. Bioinformatics.

[27] Katoh K, Rozewicki J, Yamada KD (2019). MAFFT online service: multiple sequence alignment, interactive sequence choice and visualization. Brief Bioinform.

[28] Kuraku S, Zmasek CM, Nishimura O, Katoh K (2013). aLeaves facilitates on-demand exploration of metazoan gene family trees on MAFFT sequence alignment server with enhanced interactivity. Nucleic Acids Res.

[29] Grantham R (1974). Amino acid difference formula to help explain protein evolution. Science.

[30] Tavtigian S, Greenblatt MS, Lesueur F, Byrnes GB, IARC Unclassified Genetic Variants Working Group (2008). In silico analysis of missense substitutions using sequence-alignment based methods. Hum Mutat.

[31] Castresana J (2000). Selection of conserved blocks from multiple alignments for their use in phylogenetic analysis. Mol Biol Evol.

[32] Talavera G, Castresana J (2007). Improvement of phylogenies after removing divergent and ambiguously aligned blocks from protein sequence alignments. Syst Biol.

[33] Stamatakis A (2014). RAxML version 8: a tool for phylogenetic analysis and post-analysis of large phylogenies. Bioinformatics.

[34] Drummond AJ, Rambaut A (2007). BEAST: Bayesian evolutionary analysis by sampling trees. BMC Evol Biol.

[35] Miller MA, Pfeiffer W, Schwartz T (2010). Creating the CIPRES Science Gateway for inference of large phylogenetic trees. 2010 Gateway Computing Environments Workshop (GCE).

[36] Darriba D, Taboada GL, Doallo R, Posada D (2012). jModelTest 2: more models, new heuristics and parallel computing. Nat Methods.

[37] Bandelt HJ, Forster P, Rohl A (1999). Median-joining networks for inferring intraspecific phylogenies. Mol Biol Evol.

[38] Excoffier L, Lischer HEL (2010). Arlequin suite ver 3.5: a new series of programs to perform population genetics analyses under Linux and Windows. Mol Ecol Resour.

[39] Kinkar L, Korhonen PK, Cai H, Gauci CG, Lightowlers MW, Saarma U (2019). Long-read sequencing reveals a 4.4 kb tandem repeat region in the mitogenome of *Echinococcus granulosus* (sensu stricto) genotype G1. Parasit Vectors.

[40] Baradaran R, Berrisford JM, Minhas GS, Sazanov LA (2013). Crystal structure of the entire respiratory complex I. Nature.

[41] Parey K, Haapanen O, Sharma V, Köfeler H, Züllig T, Prinz S (2019). High-resolution cryo-EM structures of respiratory complex I: mechanism, assembly, and disease. Sci Adv.

[42] Cabrera-Orefice A, Yoga EG, Wirth C, Siegmund K, Zwicker K, Guerrero-Castillo S (2018). Locking loop movement in the ubiquinone pocket of complex I disengages the proton pumps. Nat Commun.

[43] Yanagida T, Sako Y, Nakao M, Nakaya K, Ito A (2012). Taeniasis and cysticercosis due to *Taenia solium* in Japan. Parasit Vectors.

[44] Hotomo AW, Theodorus D, Veriswan I (2021). Neurocysticercosis (NCC) in 15-year-old girl, East Nusa Tenggara, Indonesia: a case report. Am J Pediatr.

[45] Sutisna P, Kapti IN, Wandra T, Dharmawan NS, Swastika K, Raka Sudewi AA (2019). Towards a cysticercosis-free tropical resort island: a historical overview of taeniasis/cysticercosis in Bali. Acta Trop.

[46] Pereira L, Macaulay V, Torroni A, Scozzari R, Prata MJ, Amorim A (2001). Prehistoric and historic traces in the mtDNA of Mozambique: insights into the Bantu expansions and the slave trade. Ann Hum Genet.

[47] Bowser F (1974). The African slave in colonial Peru, 1524–1650.

