# Supplementary material for: Assembly and phylogeographical analysis of novel Taenia solium mitochondrial genomes suggest stratification within the African-American genotype
Source: Parasit Vectors. 2023 Oct 6;16:349. doi: 10.1186/s13071-023-05958-z (PMC10559519; doi:10.1186/s13071-023-05958-z)
Supplement: Supplementary file 1 — Additional file 1: Table S1. Mapping statistics of the genome assembly. The number of reads mapped to the Chinese mitochondrial genome (reference) and their mean quality scores before trimming (pre) are given. The number of reads mapped after trimming (post); and the coverage, the genome length in base pairs (bp), the %GC, and the N50 of the assembly in nucleotides (nt) are also shown. Table S2. Gene arrangement of T. solium mitochondrial genomes from Peru, Mexico, and China (reference). The size of each genome in base pairs (bp) is given in parenthesis next to the mitochondrial genome name. Position intervals per gene are shown. The size of each gene (in bp) is specified in parentheses next to each position interval. Start and stop codons of each protein-coding gene per mitochondrial genome are also specified. Table S3. Sequence composition of the haplotypes formed in the COX1 and CYTB networks. Sequences (with their accession numbers) included in each haplotype are specified. [file 13071_2023_5958_MOESM1_ESM.docx]

| Table S1. Mapping statistics of the genome assembly. | | | |
| --- | --- | --- | --- |
| **Sample** | **Peru - Puno** | **Peru - Huancayo** | **Mexico** |
| Reads mapped (pre) | 1,376,873 | 6.272 | 683.358 |
| Mean quality score (pre) | > Q30 | > Q30 (until pos. 240) | > Q35 |
| Reads mapped (post) | 1,317,941 | 5.561 | 674.666 |
| Coverage | 7,810.92 X | 41.57 X | 3,395.09 X |
| Genome length | 13,706 bp | 13,700 bp | 13,706 bp |
| %GC | 29.23 | 30.79 | 28.54 |
| N50 | 386 nt | 482 nt | 13,651 nt |

| Table S2. Gene arrangement of *T. solium* mitochondrial genomes from Peru, Mexico, and China (reference). | | | | | | | | |
| --- | --- | --- | --- | --- | --- | --- | --- | --- |
| **Gene** | **Positions and nt size (bp)** | | | | **Start/Stop codon** | | | |
|  | Peru - Puno (13,706 bp) | Peru - Huancayo (13,700 bp) | Mexico (13,706 bp) | China (13,709 bp) | Peru - Puno | Perú - Huancayo | Mexico | China |
| **ATP6** | 5,794 - 6,309 (516) | 5,789 – 6,304 (516) | 5,794 - 6,309 (516) | 5,793 - 6,308 (516) | ATG/TAA | ATG/TAA | ATG/TAA | ATG/TAA |
| **COX1** | 9,130 - 10,749 (1,620) | 9,125 – 10,744 (1620) | 9,130 - 10,749 (1,620) | 9,129 - 10,748 (1,620) | ATG/TAG | ATG/TAG | ATG/TAG | ATG/TAG |
| **COX2** | 12,575 - 13,156 (582) | 12,566 – 13,147 (582) | 12,575 - 13,156 (582) | 12,575 - 13,156 (582) | ATG/TAG | ATG/TAG | ATG/TAG | ATG/TAG |
| **COX3** | 2,296 - 2,940 (645) | 2,294 – 2,938 (645) | 2,296 - 2,940 (645) | 2,295 - 2,939 (645) | ATG/TAG | ATG/TAG | ATG/TAG | ATG/TAG |
| **CYTB** | 3,023 - 4,090 (1,068) | 3,021 – 4,085 (1065) | 3,023 - 4,090 (1,068) | 3,022 - 4,089 (1,068) | ATG/TAA | ATG/TAA | ATG/TAA | ATG/TAA |
| **l – rRNA** | 10,805 - 11,783 (979) | 10,800 – 11,776 (977) | 10,805 - 11,783 (979) | 10,804 - 11,783 (980) |  |  |  |  |
| **s - rRNA** | 11,846 - 12,550 (705) | 11,839 – 12,541 (703) | 11,846 - 12,550 (705) | 11,846 - 12,550 (705) |  |  |  |  |
| **ND1** | 7,448 - 8,339 (892) | 7,443 – 8,334 (892) | 7,448 - 8,339 (892) | 7,447 - 8,338 (892) | ATG/T | ATG/T | ATG/T | ATG/T |
| **ND2** | 6,312 - 7,193 (882) | 6,307 – 7,188 (882) | 6,312 - 7,193 (882) | 6,311 - 7,192 (882) | ATG/TAA | ATG/TAA | ATG/TAA | ATG/TAA |
| **ND3** | 8,636 - 8,983 (348) | 8,631 – 8,978 (348) | 8,636 - 8,983 (348) | 8,635 - 8,982 (348) | ATG/TAG | ATG/TAG | ATG/TAG | ATG/TAG |
| **ND4** | 4,374 - 5,585 (1,212) | 4,369 – 5,580 (1212) | 4,374 - 5,585 (1,212) | 4,373 - 5,584 (1,212) | GTG/TAA | GTG/TAA | GTG/TAA | GTG/TAG |
| **ND4L** | 4,105 - 4,365 (261) | 4,100 – 4,360 (261) | 4,105 - 4,365 (261) | 4,104 - 4,364 (261) | ATG/TAA | ATG/TAA | ATG/TAA | ATG/TAA |
| **ND5** | 467 - 2,035 (1,569) | 467 – 2035 (1569) | 467 - 2,035 (1,569) | 468 - 2,036 (1,569) | ATG/TAA | ATG/NNN | ATG/TAA | ATG/TAA |
| **ND6** | 13,243 - 13,695 (453) | 13,234 – 13,686 (453) | 13,243 - 13,695 (453) | 13,243 - 13,695 (453) | ATG/TAA | ATG/TAA | ATG/TAA | TGT/TAG |
| **NCR 1** | 64 - 131 (68) | 64 – 131 (64) | 64-131 (68) | 64 - 131 (68) |  |  |  |  |
| **NCR 2** | 196 - 254 (59) | 196 - 254 (59) | 196-254 (59) | 196 - 254 (59) |  |  |  |  |
| **NCR 3** | 2,037 - 2,226 (190) | 2037 – 2240 (204) | 2,036-2,226 (191) | 2,037 - 2,228 (192) |  |  |  |  |
| **tRNA-Ala** | 7,292 - 7,357 (66) | 7,287 – 7,352 (66) | 7,292 - 7,357 (66) | 7,291 - 7,356 (66) |  |  |  |  |
| **tRNA-Arg** | 409 - 465 (57) | 409 – 465 (57) | 409 - 465 (57) | 410 - 466 (57) |  |  |  |  |
| **tRNA-Asn** | 8,340 - 8,408 (69) | 8,335 – 8,403 (69) | 8,340 - 8,408 (69) | 8,339 - 8,407 (69) |  |  |  |  |
| **tRNA-Asp** | 7,365 - 7,431 (67) | 7,360 – 7,426 (67) | 7,365 - 7,431 (67) | 7,364 - 7,430 (67) |  |  |  |  |
| **tRNA-Cys** | 11,784 - 11,845 (62) | 11,777-11,838 (62) | 11,784 - 11,845 (62) | 11,784 - 11,845 (62) |  |  |  |  |
| **tRNA-Gln** | 5,595 - 5,659 (65) | 5,590 – 5,654 (65) | 5,595 - 5,659 (65) | 5,594 - 5,658 (65) |  |  |  |  |
| **tRNA-Gly** | 2,230 - 2,292 (63) | 2,228 – 2,290 (63) | 2,230 - 2,292 (63) | 2,229 - 2,291 (63) |  |  |  |  |
| **tRNA-Glu** | 13,168 - 13,235 (68) | 13,159 – 13,226 (68) | 13,168 - 13,235 (68) | 13,168 - 13,235 (68) |  |  |  |  |
| **tRNA-His** | 2,945 - 3,019 (75) | 2,943 – 3,017 (75) | 2,945 - 3,019 (75) | 2,944 - 3,018 (75) |  |  |  |  |
| **tRNA-Ile** | 8,489 - 8,554 (66) | 8494 – 8559 (66) | 8,489 - 8,554 (66) | 8,488 - 8,553 (66) |  |  |  |  |
| **tRNA-Leu** | 132 - 195 (64) | 132 – 195 (64) | 132 - 195 (64) | 132 - 195 (64) |  |  |  |  |
| **tRNA-Leu** | 329 - 392 (64) | 330 – 393 (64) | 329 - 392 (64) | 330 - 393 (64) |  |  |  |  |
| **tRNA-Lys** | 8570 - 8635 (66) | 8,565 – 8,630 (66) | 8,570 – 8,635 (66) | 8,569 – 8,634 (66) |  |  |  |  |
| **tRNA-Met** | 5,718 - 5,786 (69) | 5,713 – 5,781 (69) | 5,718 - 5,786 (69) | 5,717 - 5,785 (69) |  |  |  |  |
| **tRNA-Phe** | 5,659 - 5,721 (63) | 5,654 – 5716 (63) | 5,659 - 5,721 (63) | 5,658 - 5,720 (63) |  |  |  |  |
| **tRNA-Pro** | 8,423 - 8,487 (65) | 8,418 – 8,482 (65) | 8,423 - 8,487 (65) | 8,422 - 8,486 (65) |  |  |  |  |
| **tRNA-Thr** | 10,740 - 10,804 (65) | 10,735 – 10,799 (65) | 10,740 - 10,804 (65) | 10,739 - 10,803 (65) |  |  |  |  |
| **tRNA-Trp** | 9,057 - 9,126 (70) | 9,052 – 9,121 (70) | 9,057 - 9,126 (70) | 9,056 - 9,125 (70) |  |  |  |  |
| **tRNA-Tyr** | 1 – 63 (63) | 1 – 63 (63) | 1 - 63 (63) | 1 - 63 (63) |  |  |  |  |
| **tRNA-Ser** | 254 - 313 (60) | 254 – 313 (60) | 254 - 313 (60) | 255 - 314 (60) |  |  |  |  |
| **tRNA-Ser** | 8,987 - 9,045 (59) | 8,982 – 9,040 (59) | 8,987 - 9,045 (59) | 8,986 - 9,044 (59) |  |  |  |  |
| **tRNA-Val** | 7,215 - 7,279 (65) | 7,210 – 7,274 (65) | 7,215 -7,279 (65) | 7,214 - 7,278 (65) |  |  |  |  |

| Table S3. Sequence composition of the haplotypes formed in the COX1 and CYTB networks. | | |
| --- | --- | --- |
| **Haplotype** | **COX1 sequences** | **CYTB sequences** |
| H1 | Thailand (AB066487.1) | Peru - Huancayo (From the present article),Peru - Puno (From the present article), Mexico - Mexico State 3 (FN995663.1), Mexico (AB066575.1) |
| H2 | China_3 (AB066486.1) | Indonesia - Bali, Karangasem (LC378444.1), Indonesia - Papua, Jayawijaya (LC378446.1), Indonesia - Irian Jaya (AB066573.1), Indonesia - Papua, Jayawijaya (LC378445.1), Indonesia - Bali, Karangasem (LC378443.1) |
| H3 | China_2 (AB066485.1) | India TS8 (MK059934.1), India TS6 (MK059936.1), India TS4 (MK059938.1), India TS2 (MK059940.1), India TS10 (MK059932.1), India TS32 (MK059910.1), India (AB781362.1) |
| H4 | Nepal - Sunsari (AB491985.1), Nepal - Kathmandu & Lalipur (AB524785.1), Nepal - Kathmandu & Lalipur (AB524784.1), Nepal - Kathmandu & Lalipur (AB524783.1), Nepal - Sunsari & Morang (AB524782.1), Nepal - Kathmandu & Lalipur (AB524781.1), Nepal - Sunsari & Morang (AB524780.1) | China CHI1 (AB066570.1) |
| H5 | Madagascar MDG2 (AB781356.1) | Nepal NEP1 (AB781746.1) |
| H6 | Nepal - Kathmandu (AB491986.1), Japan -Tokyo (AB516957.1),  Madagascar MDG1 (AB781355.1), India - Chandigarh (KC709810.1),  India - North (KC709806.1) | Vietnam Bac Kan (AY280805.1) |
| H7 | Madagascar MDG4 (AB781357.1) | China CHI2 (AB066571.1) |
| H8 | India - North (KC709809.1) | Madagascar MDG7 (AB781364.1), Ecuador (AB066576.1), Madagascar - Tulear 1 (FM958329.1), Mexico - Mexico State 2 (FN995662.1), Madagascar - Tulear 2 (FM958330.1) |
| H9 | Madagascar MDG6 (AB781359.1) | Brazil (AB066577.1) |
| H10 | Madagascar MDG5 (AB781358.1) | Cameroon - West (FN995668.1), Cameroon (AB066579.1), Cameroon - North (FN995667.1) |
| H11 | India - North (KC709808.1) | Mexico (From the present article) |
| H12 | China - Sichuan, Ganzi & Milong (AB984353.1) | Tanzania (AB066578.1) |
| H13 | China - Sichuan, Ganzi & Milong (AB984355.1) |  |
| H14 | China - Sichuan, Ganzi & Milong (AB984354.1) |  |
| H15 | China - Sichuan, Ganzi & Milong (AB984356.1) |  |
| H16 | India - North (KC709811.1) |  |
| H17 | China (GQ402327.1) |  |
| H18 | Indonesia - Irian Jaya (AB066488.1) |  |
| H19 | Indonesia - Baili, Karangasem (AB631045.1) |  |
| H20 | Tanzania (AB066493.1), Mexico - Yucatan (FN995660.1), Mexico - Mexico State 2 (FN995658.1), Mexico - Mexico State 1 (FN995657.1), Tanzania - Mbulu (AY211880.1) |  |
| H21 | Ecuador (AB066491.1) |  |
| H22 | Madagascar MDG7 (AB781360.1) |  |
| H23 | Madagascar MDG8 (AB781361.1) |  |
| H24 | Brazil (AB066492.1) |  |
| H25 | Peru - Huancayo (From the present article), Mexico (From the present article), Peru - Puno (From the present article), Mexico (AB066490.1), Cameroon - West (FN995666.1), Cameroon - North (FN995665.1), Mexico - Mexico State 3 (FN995659.1) |  |
